# Effects of gene polymorphisms of metabolic enzymes on the association between red and processed meat consumption and the development of colon cancer; a literature review

**DOI:** 10.1017/jns.2018.17

**Published:** 2018-10-02

**Authors:** S. Doaei, M. Hajiesmaeil, A. Aminifard, S. A. Mosavi-Jarrahi, M. E. Akbari, M. Gholamalizadeh

**Affiliations:** 1 Natural Products and Medicinal Plants Research Center, North Khorasan University of Medical Sciences, Bojnurd, Iran; 2 Cancer Research Center (CRC), Shahid Beheshti University of Medical Sciences, Tehran, Iran; 3 Department of Biology, Parand Branch, Islamic Azad University, Parand, Iran; 4 Food Sciences and Industry, Khouzestan Sciences and Research Branch, Islamic Azad University, Khouzestan, Iran; 5 Faculty of Medical School, Shahid Beheshti University of Medical Sciences, Tehran, Iran; 6 Student Research Committee, Cancer Research Center, Shahid Beheshti University of Medical Sciences, Tehran, Iran

**Keywords:** Colon cancer, Polymorphisms, Protein, Colorectal cancer, CC, colon cancer, COX, cyclo-oxygenase, CRC, colorectal cancer, CYP, cytochrome P450, MutS, mutator S, NAT, *N*-acetyltransferase, NER, nucleotide excision repair, XP, xeroderma pigmentosum

## Abstract

The role of environmental factors and genetic susceptibility in the development of colon cancer (CC) has been already proven, but the role of gene polymorphisms in modifying the risk of environmental factors such as nutritional factors is still unknown. This study aimed to investigate the effect of polymorphisms of involved genes in the association between red meat consumption and the development of CC. The present review was carried out using keywords such as polymorphism and/or protein and/or red meat and/or processed meat and/or colon cancer. PubMed and Science Direct databases were used to collect all related articles published from 2001 to 2017. The presence of SNP in the coding genes of proteins involved in metabolism of nutrients could play significant roles in the extent of the effects of nutrition in the development of CC. The effect of dietary proteins greatly depends on the polymorphisms in the metabolising genes of these substances. Gene polymorphisms may have a role in colorectal cancer risk, especially in people with high meat intake, and this leads to a difference in the effects of meat consumption in different individuals. To conclude, dietary recommendations for the prevention and control of CC should be modified based on the genotype of different individuals. Increasing our knowledge on this field of nutritional genomics can lead to personalised preventive and therapeutic recommendations for CC patients.

Colon cancer (CC), also known as colorectal cancer (CRC), is the third most commonly diagnosed malignancy and the fourth leading cause of cancer-related deaths worldwide^(^^1^^)^. The prevalence of CC among Iranian people was between 7 and 8 per 100 000 people, with a significant increase over the last several years^(^^2^^)^.

In most cases, CC occurs in people aged 50 years or older and the risk of CC recurrence is increased with age^(^^3^^)^. It has been reported that about 6 to 7 % of CC cases have a genetic origin. Approximately 10 to 15 % of CRC occur in patients where at least one of his/her relatives also had CC^(^^4^^)^. Also, some hereditary syndromes are also effective on the risk of CC including Lynch syndrome and familial adenomatous polyposis syndrome^(^^5^^)^. In addition, some environmental factors such as alcohol consumption, smoking, physical inactivity, high-fat diet and consumption of red and processed meat are also considered as risk factors for CRC^(^^3^^)^. Recent studies reported that change in the expression level of some genes is also a mechanism involved in the effects of these environmental factors^(^^6^^–^^8^^)^. Moreover, some people are at higher risk for CC because of their genotype^(^^9^^)^. In other words, the development of CRC is a complex process that involves positive and negative interactions between genes and environmental factors. In the present study, the effects of the interactions between gene polymorphisms and red and processed meat consumption on the risk of CC have been reviewed.

## Red and processed meat and colon cancer

Many studies have shown that there is a significant association between a red and processed meat-rich diet and CRC^(^^10^^,^^11^^)^. This association has been attributed to several dietary factors, including heterocyclic amines, aromatic hydrocarbons produced during high temperature heating processes, *N*-nitrosamines that are found in many food products after nitrite addition and processed meat that contains high levels of preservatives. The polymorphisms in some genes involved in the metabolism of these components and risk of CC are discussed below.

### *N*-acetyltransferases

Many studies have examined the enzymes involved in the metabolism of amines and heterocyclic amines and suggested a significant relationship between polymorphisms of these enzymes and risk of CC^(^^12^^,^^13^^)^. Heterocyclic amines are produced during cooking meat at high temperatures. *N*-acetyltransferases (NAT) are important enzymes in the metabolic activation of heterocyclic amines, which are found in two forms of NAT1 and NAT2. The rs1495741 polymorphism of *NAT2* was strongly related to its activity and the GG, AG and AA genotypes are classified as enzymes with rapid, intermediate and slow activity, respectively. In people with the GG genotype of this polymorphism, there is a strong association between the consumption of red meat and the risk of CRC^(^^12^^–^^14^^)^. Another study reported that cooking meat at a high temperature increased the risk of CC in people with *NAT2* gene polymorphisms^(^^15^^)^. However, Barrett *et al*.^(^^16^^)^ provide no support for the hypothesis that those with the fast phenotype of NAT2 are at increased risk of CRC.

A study was conducted on 147 CRC patients (seventy-six women and ninety men); the cancer risk in women was found to be lower in the NAT intermediate activity phenotype, but this difference was not found in men. It has also been reported that in people with the GG genotype of *NAT2* G857A, meat intake more than three times per week increased CRC risk^(^^17^^)^. However, some other studies failed to find any interaction between GG genotype, meat intake and CRC^(^^18^^–^^20^^)^. For example, Chan *et al*.^(^^20^^)^ reported that there was no interaction between the amount of meat consumed with NAT1 and NAT2 and the risk of developing CRC. Overall, it can be concluded that *NAT2* gene polymorphisms may have a role in CRC risk, especially in people with high meat intake.

### Cyclo-oxygenases

Cyclo-oxygenases (COX) play a key role in converting arachidonic acid into prostaglandins. Red meat contains a substantial amount of arachidonic acid and most probably is involved in the inflammatory response and initiation of CC especially in people with a polymorphism in the *COX-1* and *COX-2* genes. This polymorphism occurs in the promoter region of the gene, resulting in a possible increase in gene expression with consequent elevation of levels of the COX-2 protein. Individuals who carry the polymorphisms that could affect the expressions of *COX-2* are more susceptible to CC^(^^21^^)^. There are two isoforms of the COX enzyme, COX-1 (or prostaglandin-endoperoxide synthase 1; PTGS1), that produces PG1, and COX-2 (or PTGS2), which produces PG2. The rs20417 (−765G > C) and rs5275 (8473T > C) polymorphisms of *COX-2* play an important role in many cancers such as gastric cancer, prostate cancer and CRC. Some studies have also shown that the *COX-2* rs1195AA genotype can also play a supportive role in the development of CRC. Makar *et al*.^(^^22^^)^ showed that polymorphism rs20417 (−765G > C) in the *COX-2* gene increases the risk of rectal cancer by up to two times higher than others. No significant relationship was reported between *COX-1* gene polymorphisms and CRC in this study. In one meta-analysis study, there was a significant relationship between the *COX-2* rs20417 polymorphism and the risk of CRC in an Asian population^(^^23^^)^. Andersen *et al*.^(^^24^^)^ suggested that the relationship between the *COX-2* rs20417 polymorphism and the risk of CRC is influenced by dietary meat intake and *COX-2* rs20417 risk allele carriers were at 8 % increased risk of CRC per 25 g/d higher red meat or processed meat intake. Generally, it can be concluded that *COX-2* gene polymorphisms may have a role in CRC risk, especially in people with higher meat intake.

### Cytochrome P450 2E1 and cytochrome P450 1A2

CRC is associated with environmental factors such as cigarette smoking, and consuming cooked meats and fish at high temperature. These factors result in the formation of carcinogenic compounds including polycyclic aromatic hydrocarbons, arylamines and heterocyclic amines. The cytochrome P450 (CYP) enzymes are critically important for the metabolism of these carcinogens by N oxidation^(^^25^^)^. CYP2E1 is an enzyme that plays a key role in the metabolism of nitrosamines and other carcinogens^(^^26^^)^. The *Rsa*I polymorphism of *CYP2E1* (C2 allele) is associated with an increased risk of CRC^(^^26^^,^^27^^)^. The *Rsa*I polymorphism has been shown to affect its transcription level. The variant type of this polymorphic site can enhance transcription and increase the level of CYP2E1 enzymic activity *in vitro*^(^^10^^)^.

Some studies have also shown that individuals carrying a variant of the C2 allele have lower enzyme activity. In the Hawaiian population, it has been shown that the risk of CC has decreased in subjects carrying the *Rsa*I C2 allele^(^^27^^)^. On the other hand, a study in China showed that homozygous individuals for the C2 allele were also more likely to develop CRC^(^^28^^)^. Moreover, in some other studies, no relationship was observed between *Rsa*I polymorphisms and the risk of CRC^(^^29^^)^. Interestingly, Morita *et al*.^(^^30^^)^ showed that there is a significant relationship between red meat consumption and an increased risk of CC in the individuals carrying the *Rsa*I C2 allele. However, another study reported that no significant relationship was observed between the the *CYP2E1 Rsa*I polymorphism, red meat consumption and CC^(^^31^^)^.

*CYP1A2*, a member of the cytochrome P450 mixed-function oxidase system, is involved in the metabolism of xenobiotics in the body^(^^32^^)^. Some studies have shown that individuals carrying *CYP1A2* polymorphisms are at higher risk of developing rectal cancer but not for CC^(^^33^^–^^35^^)^. In a case–control study on *CYP1A2* polymorphisms, it was found that there was a significant relationship between the consumption of cooked meat at high temperature in −154A > C polymorphism carriers of *CYP1A2* and the risk of CRC. Overall, it is possible that *CYP2E1* and *CYP1A2* gene polymorphisms may have a role in CRC risk, especially in people with higher meat intake.

### Nucleotide excision repair pathway

The nucleotide excision repair (NER) pathway plays an important role in repairing damaged DNA. The NER pathway is a particularly important excision mechanism that removes DNA damage induced by UV light and environmental carcinogens^(^^36^^)^. Xeroderma pigmentosum (XP) complementation group A (XPA), XP complementation group C (XPC) and XP complementation group D (XPD) are important enzymes in the NER pathway. There is a significant relationship between polymorphisms in *XPA*, *XPC* and *XPD* and a lower capacity of DNA repair. Numerous polymorphisms of *NER* genes have been identified and these changes individually or in combination may adversely affect NER fidelity, which could contribute to the risk of CRC. Four polymorphisms of these genes including A23G in *XPA*, Lys939Gln in *XPC*, and Lys751Gln and Asp312Asn in *XPD* have been identified that may have a significant relationship with the risk of CC^(^^37^^)^. For example, in a study conducted by Hansen *et al*.^(^^38^^)^, a lower risk of cancer was reported in women with the Lys751Gln polymorphism of *XPD*. In homozygous individuals with the *XPC* Lys939Gln polymorphism, the risk of CC was increased by 3·7 times per 100 g/d increased intake of red meat. In the individuals carrying the wild-type allele, meat has no effect on CRC. No significant relationship was observed between other polymorphisms and CC.

Moreover, it was shown that people with a high consumption of red meat and *XPD* 312Asp and *XPD* 751Lys risk alleles have a higher chance of developing CRC than those with *XPD* 312Asn and *XPD* 751Gln alleles^(^^39^^)^. There is also a statistically significant interaction between Lys939Gln of *XPC* and A23G of *XPA* with red meat and processed meat intake and the risk of CC^(^^38^^,^^40^^)^. Overall, it can be concluding that higher meat intake may have a role in CRC risk, especially in people with polymorphisms in genes involved in the NER pathway.

### DNA mismatch repair (mutator S)

A DNA mismatch repair protein, also known as mutator S (MutS), participates in the DNA mismatch repair system. In a study conducted on the polymorphisms of this gene, it was found that some gene polymorphisms were associated with an increased risk of CC. Processed meat intake could increase CC risk in people with the *MutS* polymorphism^(^^41^^)^. In another study, a significant relationship was observed between processed meat intake, the – polymorphism of the *MutS* gene and the risk of CC^(^^42^^)^. In general, it can be concluded that *MutS* polymorphisms may have a role in CRC risk, especially in people with higher processed meat intake.

## Discussion

The presence of SNP associated with the metabolism and function of proteins could play an important role in the effects of red meat consumption on the risk of CC.

Several individual SNP have been associated with CC risk. It is plausible that a set of SNP derived from genetic pathways that are critical in colon carcinogenesis could contribute to the cancer risk. We investigated the role of polymorphisms involved in five metabolic pathways that are relevant for the activation or detoxification of carcinogens formed during red meat processing. The polymorphisms investigated in the present study were mostly functional polymorphisms that alter the expression of genes participating in metabolic pathways associated with carcinogenesis^(^^43^^)^.

Recent studies demonstrated the modifier role of *NAT2* G857A, *COX-2* rs20417, *CYP2E1 Rsa*I, *CYP1A2* 154A>C, *XPC* Lys939Gln, *XPA* A23G and *MutS* T1036A on the effect of red meat consumption on CRC risk. However, some studies failed to identify an association between red meat consumption and the effect of these polymorphisms on CRC risk. Possible explanations for the discrepancy might include differences in meat variable definitions, and lack of stratification by tumour subsite in these studies. Moreover, other factors including frequency of turning the meat over during the cooking process, meat thickness, cut of meat, use of marinade or thawing meat in the microwave were not considered and may have contributed to these contradictory results^(^^44^^)^.

## Conclusion

In conclusion, some gene polymorphisms may have a significant role in CRC risk, especially in people with higher processed meat intake. Increasing the knowledge on nutritional genomics can lead to the finding of new methods to prevent, treat and control of CC. A summary of descriptions of studies is presented in Table 1.

**Table 1.** Summary of study descriptions and outcomes

Reference	Title	Study design	Sample characteristic	Examined components	Main findings
NAT					
Wang *et al*. (2015)^(^^12^^)^	Interaction between red meat intake and *NAT2* genotype in increasing the risk of CRC in Japanese and African Americans	Meta-analyses	2744 cases, 8315 controls	*NAT* genotype (SNP rs1495741)	In people with GG genotype (rapid NAT2 phenotype) of this polymorphism, there is a strong association between consumption of red meat and the risk of CRC
Ananthakrishnan *et al*. (2015)^(^^13^^)^	Red meat intake, *NAT2*, and risk of CRC: pooled analysis of 11 studies	Pooled analysis	8290 cases, 9115 controls	NAT2 phenotype based on polymorphism at rs1495741	High red meat consumption was similarly associated with CRC in those with a rapid/intermediate *NAT2* genotype
Barrett *et al*. (2003)^(^^16^^)^	Investigation of interaction between *NAT2* and HA as potential risk factors for CRC	Case–control study	484 cases, 738 controls	NAT2 phenotype	This study provides no support for the hypothesis that fast NAT2 acetylators are at increased risk of CRC, even if exposed to high levels of HA from well-cooked meat or smoking
Sørensen *et al*. (2008)^(^^14^^)^	Prospective study of *NAT1* and *NAT2* polymorphisms, tobacco smoking and meat consumption and risk of CRC	Case–control study	379 cases, 769 controls	NAT 1 and NAT2 fast and slow NAT acetylator phenotypes	There were statistically significant associations between consumption of brown-dark pan-fried meat and increased CRC risk. NAT1 fast acetylators had a significantly higher risk of CRC than NAT1 slow acetylators, whereas NAT2 acetylator phenotype did not affect the CRC risk
Lilla *et al*. (2006)^(^^15^^)^	Effect of *NAT1* and *NAT2* genetic polymorphisms on CRC risk associated with exposure to tobacco smoke and meat consumption	Case–control study	505 patients with incident CRC, 604 controls	NAT 1 and NAT2 fast and slow NAT acetylator phenotypes	Cooking meat at high temperature increased the risk of CC in people with *NAT2* gene polymorphisms
Procopciuc *et al*. (2017)^(^^17^^)^	*NAT2*/environmental factors and their association as a modulating risk factor for sporadic colon and rectal cancer	Case–control study	150 cases, 162 controls	NAT2 phenotypes	Fried red meat, alcohol and smoking increase the risk of sporadic CRC, especially of colon cancer, in the case of rapid acetylators for the NAT2 variants
Da Silva *et al*. (2011)^(^^18^^)^	*NAT2* genetic polymorphisms and risk of CRC	Case–control study	147 patients with CRC, 162 controls	People with GG genotype (NAT2 fast acetylators)	Among NAT2 fast acetylators, meat intake more than three times per week increased the risk of CRC
Tiemersma *et al*. (2002)^(^^19^^)^	Meat consumption, cigarette smoking and genetic susceptibility in the aetiology of CRC	Case–control study	102 incident CRC cases, 537 controls	*NAT2* gene polymorphisms (NAT2 fast acetylators)	This study found no association between GG genotype and CRC
Chan *et al*. (2005)^(^^20^^)^	Prospective study of *NAT2* genotypes, meat intake, smoking and risk of CRC	Nested case–control study	183 women with CRC, 443 controls	*NAT2* gene polymorphisms	This study found no interaction between meat consumption with *NAT2* and CRC
COX					
Zhu *et al*. (2010)^(^^45^^)^	−2765G>C and 8473T > C polymorphisms of *COX-2* and cancer risk: a meta-analysis based on 33 case–control studies	Meta-analyses	19 100 cases, 29 777 controls	*COX-2* gene, −2765G > C and 8473T > C polymorphisms	This study suggested that −765G > C may cause an increased risk of colorectal carcinoma in those of Asian descent
Makar *et al*. (2013)^(^^22^^)^	*COX-1* (*PTGS1*) and *COX-2* (*PTGS2*) polymorphisms, NSAID interactions, and risk of colon and rectal cancers in two independent populations	Case–control study	2053 colon and rectal cancer patients, 2648 controls	*COX-2* gene, rs20417 (−765G> C) polymorphism	The rs20417 (−765G > C) polymorphism in the *COX-2* gene increases the risk of rectal cancer by up to two times. However, no significant relationship was reported between *PTGS1* and CRC in this study
Andersen *et al*. (2013)^(^^24^^)^	Interactions between diet, lifestyle and *IL-10*, *IL-1B* and *PTGS2*/*COX-2* gene polymorphisms in relation to risk of CRC in a prospective Danish case–cohort study	Case–control study	9070 CRC cases, 1789 controls	*COX-2* gene, −765G>C polymorphism	Suggested that *COX-2* −765G < C risk allele carriers were at 8 % increased risk of CRC per 25 g red meat or processed meat intake per d
*CYP2E1*					
Le Marchand *et al*. (2002)^(^^10^^)^	Red meat intake, *CYP2E1* genetic polymorphisms, and CRC risk	Case–control study	521 patients with CRC, 639 controls	Polymorphisms in *CYP2E1*	This study showed that individuals carrying a variant of the C2 allele have lower enzyme activity
van der Logt *et al*. (2006)^(^^28^^)^	Role of epoxide hydrolase, NAD(P)H:quinone oxidoreductase, *CYP2E1* or alcohol dehydrogenase genotypes in susceptibility to CRC	Case–control study	371 patients with sporadic CRC, 415 healthy controls	Polymorphisms in *CYP2E1*	Homozygous individuals for the C2 allele were also more likely to develop CRC
Morita *et al*. (2009)^(^^30^^)^	Genetic polymorphisms of *CYP2E1* and risk of CRC: the Fukuoka Colorectal Cancer Study	Case–control study	685 incident cases of CRC, 778 controls	Polymorphisms in *CYP2E1*	Risk of cancer in individuals carrying *CYP2E1 Rsa* I C2 allele decreases in comparison with patients carrying *CYP2E1* 96-bp insertion
Wang *et al*. (2012)^(^^32^^)^	Carcinogen metabolism genes, red meat and poultry intake, and CRC risk	Case–control study	577 cases, 307 controls	−154A>C polymorphism of *CYP1A2*	There was a significant relationship between the −154A>C polymorphism of *CYP1A2* and consumption of cooked meat at high temperature with the risk of CRC
Nucleotide excision repair pathway					
Khan *et al*. (2000)^(^^37^^)^	A new *XPC* poly(AT) insertion/deletion polymorphism	Case–control study	419 cases, 219 controls	Four polymorphisms including A23G in *XPA*, Lys939Gln in *XPC*, and Lys751Gln and Asp312Asn in *XPD*	Significant relationship with the risk of CC
Hansen *et al*. (2007)^(^^38^^)^	*XPA* A23G, *XPC* Lys939Gln, *XPD* Lys751Gln and *XPD* Asp312Asn polymorphisms, interactions with smoking, alcohol and dietary factors, and risk of CRC	Case–control study	405 CRC cases, 810 controls	*XPC* Lys939Gln, *XPA* A23G, *XPD* Lys751Gln, and *XPD* Asp312Asn polymorphisms	This study showed lower risk of cancer in women with Lys751Gln polymorphism of *XPD* and in homozygous individuals for *XPC* Lys939Gln polymorphism increased CC
Joshi *et al*. (2009)^(^^39^^)^	Red meat and poultry intake, polymorphisms in the nucleotide excision repair and mismatch repair pathways and CRC risk	Case–control study	577 cases, 307 controls	*XPD* 312ASP and *XPD* 751Lys	People with high consumption of red meat and *XPD* 312ASP and *XPD* 751Lys risk alleles have a higher chance of CRC. The consumption of poultry meat in the carriers of the *XPD* 751Lys allele increased risk of CC
Steck *et al.* (2014)^(^^40^^)^	Nucleotide excision repair gene polymorphisms, meat intake and colon cancer risk	Case–control study	331 African Americans with colon cancer, 544 controls	*XPC* (A499V and K939Q), *XPD* (D312N and K751Q), *XPF* (R415Q), *XPG* (D1104H) genotypes	This study showed the statistically significant positive association between colon cancer risk and *XPC* 499 AV + VV genotype and an inverse association with *XPC* 939 QQ
DNA mismatch repair (*MutS*)					
Berndt *et al*. (2007)^(^^41^^)^	Mismatch repair polymorphisms and the risk of CRC	Case–control study	237 CRC cases and a subcohort of 2189 participants	Four SNP in three mismatch repair genes (*MSH3* R940Q, *MSH3* T1036A, *MSH6* G39E and *MLH1* I219V) were genotyped	Processed meat intake appeared to modify the association between *MSH3* polymorphisms and CRC

NAT, *N*-acetyltransferase; CRC, colorectal cancer; HA, heterocyclic amines; COX, cyclo-oxygenase; PTGS, prostaglandin-endoperoxide synthase; NSAID, non-steroidal anti-inflammatory drugs; *CYP2E1*, cytochrome P450 2E1; *XP*, xeroderma pigmentosum; *MutS*, mutator S; *MSH*, *MutS* homolog 3; *MLH*, *MutL* homolog 1.
